# A comparative dosimetric study of left sided breast cancer after breast-conserving surgery treated with VMAT and IMRT

**DOI:** 10.1186/s13014-015-0531-4

**Published:** 2015-11-17

**Authors:** Hongfu Zhao, Mingyuan He, Guanghui Cheng, Dongmei Han, Ning Wu, Dan Shi, Zhipeng Zhao, Jianxue Jin

**Affiliations:** Department of Radiation Oncology, China-Japan Union Hospital of Jilin University, Changchun, 130033 China; Department of Radiation Physics, Elekta China Co. Ltd, Beijing, 100101 China

**Keywords:** Left sided breast cancer, IMRT, VMAT, Dosimetry

## Abstract

**Background and purposes:**

This study compared VMAT and IMRT plans for intact breast radiotherapy for left sided breast cancer and evaluated the irradiated dose of planning target volume and OARs, especially focusing on heart and coronary artery.

**Materials and methods:**

Eleven patients with left sided breast cancer whose breast was relatively smaller (the mean volumes is 296 cc) treated with breast-conserving surgery were prescribed radiotherapy of 50 Gy in 25 fractions using two or four-field step and shoot IMRT (2 or 4-F IMRT), and one or two-arc VMAT (1 or 2-arc VMAT). The 10 Gy electron boost to the tumor bed after delivery of 50 Gy was not included in the analysis. Multiple planning parameters for the PTV and the PRV-OARs were measured and analyzed.

**Results:**

Treatment plans generated using VMAT had better PTV homogeneity than the IMRT plans. For the PRV-OARs, the 1-arc VMAT had significantly higher D_mean_ and V5 for left lung and heart, and showed worse D_mean_ for liver, esophagus, spinal cord, contralateral lung and breast. In contrast, the 2-arc VMAT and the 2-F or 4-F IMRT plans showed better results for the PRV-OARs than the 1-arc VMAT. However, for the heart and coronary artery, the 1-arc VMAT showed better V20 and V40 compared with the other plans. Moreover, the 2 F-IMRT had specially advantage on V5 and V20 for heart and V5 for coronary arteries, the 2-F IMRT also showed a greater MU and treatment times. Using the table of quality score to evaluate the plans, we found that 2-F IMRT had the highest scores of 13, followed by the 2-arc VMAT plan (10 points) and 1-arc VMAT plan (8 points), and finally the 4-F IMRT plan (6 points). Moreover, when a dose comparison for heart minus coronary artery was calculated, the V20 and V40 for the rest of heart in all plans were very small and closed, indicating the dose to the coronary artery contributed dramatically to the high dose volumes for the entire heart.

**Conclusions:**

Compared to other plans, the 2-F IMRT plan with fewer monitor units and shorter delivery time is an appropriate technique for left sided breast cancer, which achieved good PTV coverage and sparing of organs at risk besides for the heart and coronary artery.

## Introduction

Breast cancer is the most common cancer among women. About 1.2 million women are newly diagnosed with breast cancer each year in the world, and 500,000 women die of it each year. Therefore, the breast cancer remains the primary cause of cancer mortality in women after lung cancer. A number of randomized controlled clinical trials have shown that breast-conserving surgery (BCS) combined with post-operative radiation therapy (PORT) [1] has the same curative effect as the Halsted radical mastectomy [2], making this the primary therapeutic strategy for Stages I and II breast cancer. PORT has been shown to substantially reduce the rate of local relapse and improve long-term survival [3] but at the cost of morbidity to the heart [4], lung [5] and a risk of secondary breast cancer [6]. Among these Organs at Risk (OARs), the heart is one of the most important, in particular in relation to radiotherapy for left-sided breast cancer where cardiac dose has been associated with increased cardiac mortality [7, 8].

Three-dimensional conformal radiation therapy (3-D CRT) and intensity-modulated radiation therapy (IMRT) techniques have been implemented across China. Traditionally, breast radiotherapy used a fluoroscopic technique with two dimensional planning. This was followed by 3-D CRT with two conventional tangential radiotherapy fields. IMRT has been widely used for the past decade, permitting variation of fluence across the radiotherapy fields, and allowing optimal dose distribution according to an individual’s anatomy. It has been suggested that IMRT results in improved dose homogeneity within the irradiated breast with added sparing of the heart and lung when compared with 3-D CRT [9, 10]. Breast IMRT ranges from photon-only IMRT to mixed electron and photon IMRT with 2 to 16 fields of various photon and electron beam energies [11]. Dogan et al*.* [12] investigated the number of beams necessary for optimal dose coverage of the breast and found that 4-field IMRT was the best choice. A newer technique known as volumetric modulated arc therapy (VMAT) was introduced in 2007 as a novel extension of IMRT, in which an optimized three-dimensional dose distribution could be delivered in a single gantry rotation. Compared to IMRT planning, VMAT resulted in even better Planning Target Volume (PTV) coverage and sparing of OARs than IMRT [13]. However, Badakhshi et al*.* argued that VMAT was inferior to IMRT and 3D-CRT with regard to dose distribution to organs at risk, especially at the low dose level, therefore VMAT was not recommended for breast cancer treatment compared with IMRT or conventional radiotherapy [14]. To further assess the advantages and the disadvantages of different IMRT and VMAT plans in whole left breast irradiation for the breast cancer patient, the VMAT (1 and 2-arc VMAT) plans and IMRT (2 and 4-field IMRT) plans were examined in a prospective clinical setting to adequately evaluate the irradiated dose analysis of planning target volume and OARs, especially focusing on heart coronary artery (CA).

## Material and methods

### Patients

Eleven patients with stage 0 (two patients), stage I (five patients) and stage II (four patients: two patients were diagnosed with N1) left-sided breast cancer were randomly selected for this treatment planning study. They had undergone breast-conserving surgery. The mean patient age at treatment was 45 years (range from 32 to 67 years). This study was approved by the Research Ethics Board of the China-Japan Union Hospital of Jilin University, and all patients agreed to the conditions of this trial. Completed informed consent forms were obtained from each patient.

### Target and normal tissue delineation

The breast Clinical Target Volume (CTV) included all visible breast parenchyma, retracted 5 mm from the skin surface. The Planning Target Volume (PTV) comprised the CTV with a 7 mm circumferential margin to allow for daily set-up variations and account for setup uncertainties and respiratory motion, and was also retracted 5 mm from the skin surface. The breast PTVs ranged from 149 cm^3^ to 537 cc (296.6 ± 122.2 cc).

The Planning Risk Volume (PRV) of all the involved OARs, including contra-lateral breast, entire heart, coronary artery area, liver, spinal cord, esophagus, left lung and right lung were outlined by the treating physician. According to the American Memorial Sloan-Kettering Cancer Research Methods (AMSK CRM), one-fourth of the left anterior aspect of the heart, up to 1 cm subsurface, is identified as the volume encompassing the coronary artery (CA) area [15].

### Planning procedure

Both VMAT and step and shoot IMRT plans were completed in the three-dimensional treatment planning system (TPS) Monaco® v.3.20.02 Elekta AB, Stockholm, Sweden). The TPS determines densities in the body based on CT density calibration curves, and calculates dose with a Monte Carlo Photon algorithm, taking into account the calibration for an inhomogeneous medium. An Elekta Synergy® (Elekta AB, Stockholm, Sweden) linear accelerator fitted with the Multi Leaf Collimator MLCi™ (1 cm leaf width) was used with a 6 MV photon energy beam. All plans were normalized to the 95 % isodose line encompassing 95 % of the PTV (V95 % = 47.5 Gy).

**Fig. 1:**
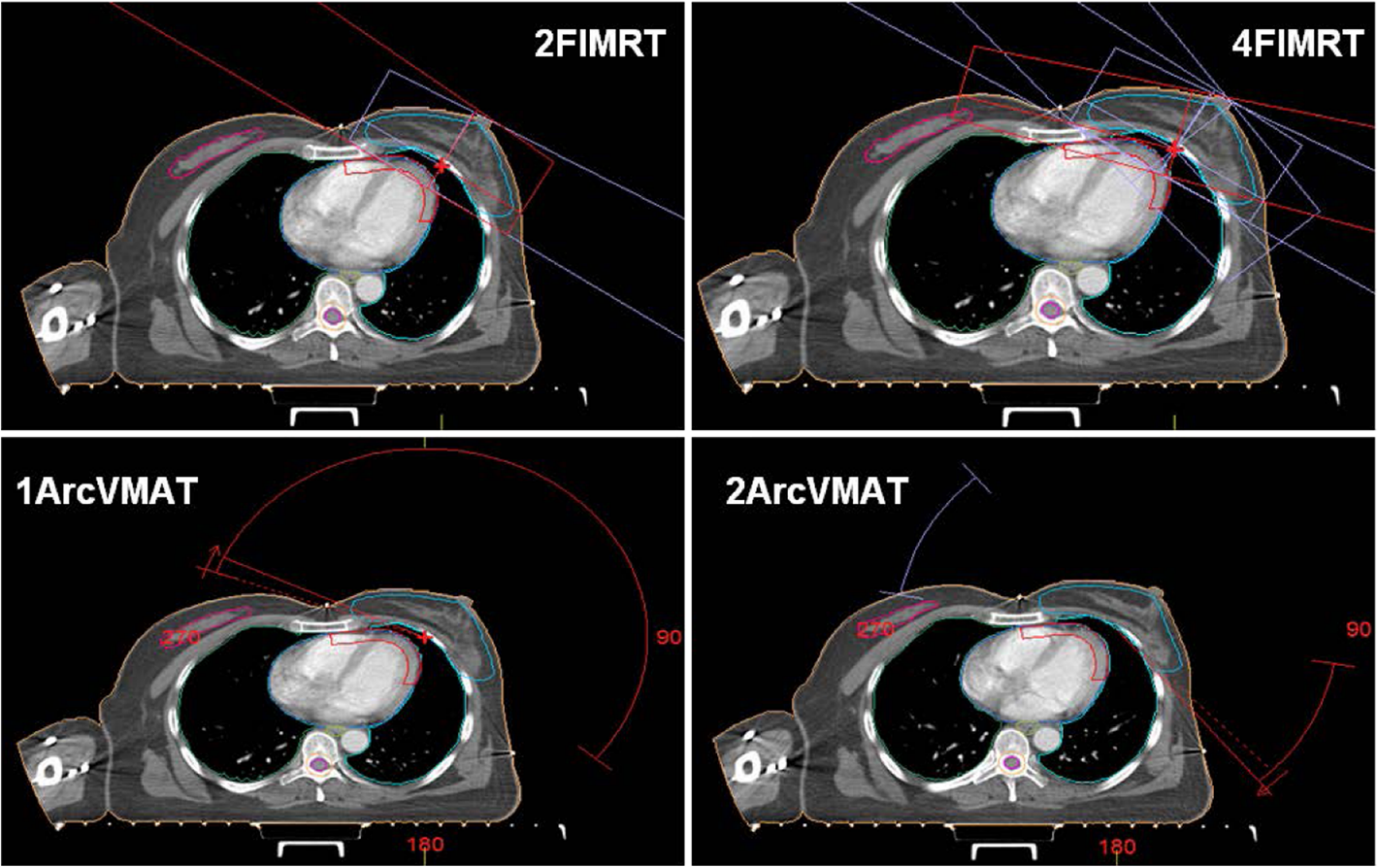
IMRT and VMAT treatment plans with corresponding examples of segment shapes and digitally reconstructed radiographs (DRRs) for left-sided breast cancer

The 2-F IMRT plan used two opposed modulated fields, which suited the PTV curved shape and orientation. The gantry angle was determined according to the angle of the curve formed by the PTV. On the basis of the 2-F IMRT, the 4-F IMRT plan added two modulated fields, thereby better avoiding the coronary artery area. A 2-arc VMAT (4 shuttle subarcs) plan was generated using two small 40°rotations (the gantry angle of middle line of the arc was identical with the direction with the 2-F IMRT and was same with it) which followed the orientation of the 2-F IMRT. The 1-arc VMAT (2 shuttle subarcs) plan was generated using a single 210°rotation with a starting angle and ending angle similar to that of the 4-F IMRT. This sector angle was used to avoid entrance doses to the contralateral lung and heart. The minimum subarc segment area was 2 cm^2^; the minimum number of monitor units was four; the maximum number of control points per arc was 60; the minimum segment width was 1 cm, and the fluence smoothing was at medium level. The four-field arrangements are shown in Fig. 1, and use the same optimization objective. Optimization prioritized normal tissue constraints, and used segment shape optimization.

### Prescribed dose

The prescription dose to the whole breast was 50 Gy in 25 fractions (D50 = 50 Gy) according to the ICRU report number 83 recommendations [16]. The prescription properties are shown in Table 1 under Monaco system. We used a 10 Gy electron boost to the tumor bed after delivery of 50 Gy to the entire breast (not included in the doses analyzed).

**Table 1:** The prescription properties of the IMRT and VMAT plans

Structure	Cost Function	Constraints
PTV	Target Penalty	Prescription:5000 cGy Minimum Volume:50 % Surface Margin:0.5 cm
	Quadratic Overdose	Maximum Dose:5150 cGy RMS Dose Excess:50 cGy Shrink Margin:0.00 cm
	Underdose DVH	Objective Dose:4750 cGy Minimum Volume:95 %
Coronary Artery	Overdose DVH	Objective Dose:500 cGy Maximum Volume:76 % Shrink Margin:0.5 cm
	Overdose DVH	Objective Dose:1000 cGy Maximum Volume:55 % Shrink MarginL:0.2 cm
	Overdose DVH	Objective Dose:2000 cGy Maximum Volume:46 % Shrink MarginL:0.1 cm
Left Lung	Maximum Dose	Maximum Dose:4950 cGy Shrink Margin:0.8 cm
	Overdose DVH	Objective Dose:500 cGy Maximum Volume:30 % Shrink Margin:0.0 cm Optimize over all voxels in volume
	Overdose DVH	Objective Dose:1000 cGy Maximum Volume:20 % Shrink Margin:0.0 cm
	Overdose DVH	Objective Dose:2000 cGy Maximum Volume:15 % Shrink Margin:0.0 cm
Heart	Overdose DVH	Objective Dose:3000 cGy Maximum Volume:3 % Shrink Margin:1.0 cm
Right Breast	Overdose DVH	Objective Dose:500 cGy Maximum Volume:4 % Shrink Margin:0.3 cm
Right Lung	Overdose DVH	Objective Dose:500 cGy Maximum Volume:6 % Shrink Margin:0.0 cm
Liver	Maximum Dose	Maximum Dose:1500 cGy Shrink Margin:0.8 cm
Esophagus	Maximum Dose	Maximum Dose:500 cGy Shrink Margin:0.0 cm
Spinal cord	Maximum Dose	Maximum Dose:800 cGy Shrink Margin:0.5 cm
Body	Quadratic Overdose	Maximum Dose:5000 cGy RMS Dose Excess:30 cGy Shrink Margin:0.00 cm
	Quadratic Overdose	Maximum Dose:4500 cGy RMS Dose Excess:40 cGy Shrink Margin:1.00 cm
	Maximum Dose	Maximum Dose:6050 cGy Shrink Margin:0.00 cm Optimize over all voxels in volume
	Maximum Dose	Maximum Dose:5600 cGy Shrink Margin:0.50 cm

### Dosimetric evaluation parameters

The following parameters were evaluated to assess plan quality: for the PTV, the dose to 98 and 2 % of the volume (D98 % and D2 %, respectively) and the part of the PTV receiving more than 107 % of the prescribed dose (V107 %) were explicitly calculated. Additionally, the Homogeneity Index (HI) was calculated according to ICRU 83 [17]: HI = (D2 %–D98 %)/D50 %. The Conformity Index (CI) as proposed by Paddick et al*.* [18] was evaluated: CI = V_47.5_/PTV. The Conformity Number (CN) = (VPTV_47.5_/V_PTV_)*(VPTV_47.5_/V_Body47.5_), and integral dose (ID) = V_Body_*D_Mean in body, outside PTV_ were also reconstructed. Total treatment delivery time was measured on the quality assurance model in phantom and recorded from end of phantom setup to the end of the treatment, not including the idle frame time. The primary field switching system could trigger automatically.

### Plans of quality analysis in population and individual case

To better summarize the most superior technique from the multi-parameter results of our study, we use the quality score table of the plans for evaluation. The parameter setting in the table was chosen according to the weight factors of our concerning in our research. In the score table, it is scored to point 1 if one parameter showed significant advance (*p* < 0.05) comparing with another parameter among the different plans, otherwise scored to 0. And only the best index/indices could get 1 point in each parameter. Moreover, we used individual plan comparison table for evaluating the technique in each parameter/patient by tabulating the number of the patients and recording or calculating the best plan (with favorable index among the four plans).

### Statistics

All results were compared and analyzed using a two-sided paired *t* test by SPSS 11.5 software, and a statistical significance level of 0.05 was used (*p* < 0.05).

## Results

### Dose analysis of planning target volume

**Fig. 2:**
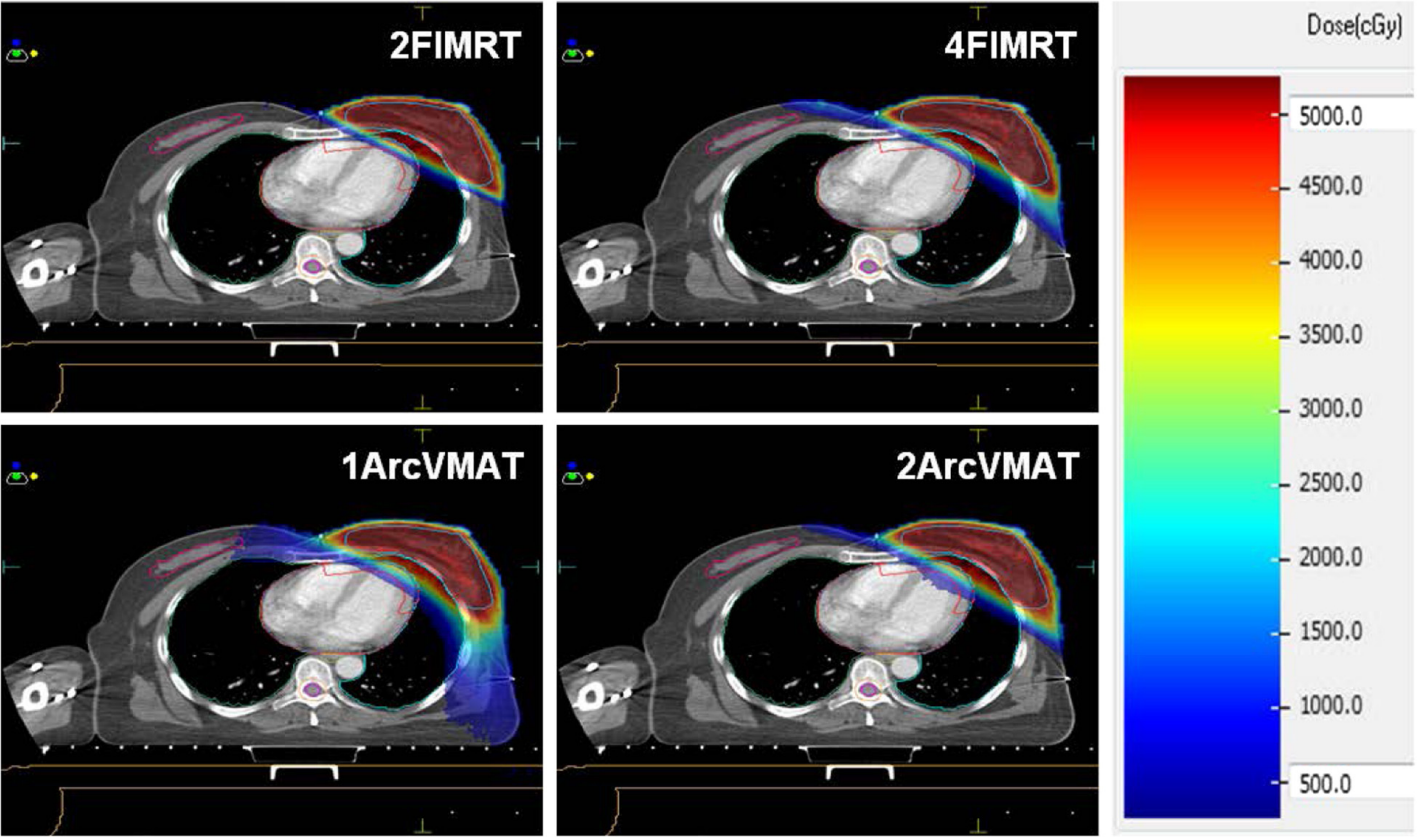
Isodose distributions for IMRT and VMAT treatment plans

Figure 2 shows axial dose distributions with VMAT and IMRT. Both VMAT and IMRT achieved 95 % coverage of the PTVs. Mean values for HI, CI and CN are presented in Table 2. We found that both VMAT and IMRT achieved good dose homogeneity across the whole breast for all patients in this study. The two VMAT plans have better HI and CI than the two IMRT plans, and decreased V107, too. However, the difference in HI and CI was not significant between any two IMRT or VMAT plans.

**Table 2:** PTV dose parameters for four plans

	2FIMRT	4FIMRT	2ArcVMAT	1ArcVMAT
D98 (Gy)	46.2 ± 0.6 ^A^	46.4 ± 0.4	46.6 ± 0.4 ^B^	46.6 ± 0.6 ^B^
D50 (Gy)	50.7 ± 0.5 ^A^	50.2 ± 0.3 ^B,a^	49.7 ± 0.5 ^B,b^	49.7 ± 0.6 ^B,b^
D2 (Gy)	53.8 ± 0.7 ^A^	53.5 ± 0.5 ^A^	52.5 ± 0.6 ^B^	52.5 ± 1.1
V107	4.6 % ± 5.4 % ^A^	2.2 % ± 1.8 % ^a^	0.6 % ± 1.1 % ^B^	0.7 % ± 0.9 % ^B,b^
HI	0.150 ± 0.022 ^A^	0.140 ± 0.016 ^A^	0.118 ± 0.018 ^B^	0.120 ± 0.029 ^B^
CI	1.34 ± 0.10 ^A^	1.32 ± 0.10 ^A^	1.24 ± 0.07 ^B,a^	1.16 ± 0.05 ^B,b^
CN	0.677 ± 0.050 ^A^	0.685 ± 0.051 ^A^	0.728 ± 0.036 ^B,a^	0.781 ± 0.032 ^B,b^
MU	265.8 ± 15.6 ^A,b^	353.1 ± 41.0 ^A,a^	494.1 ± 54.5 ^B,b^	598.3 ± 93.9 ^B,a^
Time (Second)	115.8 ± 10.0 ^A^	206.3 ± 25.0 ^B,a^	160.9 ± 17.6 ^B,b^	189.1 ± 27.0 ^B,a^

“A” is statistically significantly different from “B” (*p* < 0.05); “a” is statistically significantly different from “b” (*p* < 0.05). No other statistically significant difference was found between any two (*p* > 0.05)

### MUs and delivery time

Table 2 also shows that MUs and the estimated treatment time per 2 Gy fraction for the 2-F IMRT plan was significantly lower than other three plans. With respect to the both MUs and the treatment time, the values for 1-arc VMAT plans were the worst.

### Dose analysis of OARs

The D_mean_ in healthy tissue (outside of the PTV) increased in the plans, from the 2-F IMRT, to 2-arc VMAT, to the 4-F IMRT, to the 1-arc VMAT (Table 3).

**Table 3:** Dose comparison of the ipsilateral lung, the contralateral lung, contralateral breast, liver, esophagus, and spinal cord in the four plans

Structure	Dose parameter	2FIMRT	4FIMRT	2ArcVMAT	1ArcVMAT
Body-PTV	D_mean_(Gy)	2.2 ± 0.7 ^A^	2.4 ± 0.7 ^A^	2.3 ± 0.7 ^A^	2.8 ± 0.8 ^B^
	ID (Gy*litre)	43.7 ± 13.5 ^A^	48.0 ± 13.9 ^B,a^	44.7 ± 13.6 ^A,a^	54.5 ± 16.7 ^B,b^
Left Lung	D_mean_(Gy)	4.9 ± 2.4 ^A^	5.2 ± 2.1	5.0 ± 2.0 ^A^	5.4 ± 1.8 ^B^
	V5	16.6 % ± 7.9 % ^A^	19.1 % ± 7.7 % ^B^	18.4 % ± 7.2 % ^B,a^	21.7 % ± 6.9 % ^B,b^
	V20	9.3 % ± 5.1 %	9.5 % ± 4.7 %	9.2 % ± 4.2 %	9.3 % ± 4.1 %
	V40	4.3 % ± 2.8 % ^A^	3.7 % ± 2.3 % ^B,a^	3.8 % ± 2.2 % ^a^	3.1 % ± 1.9 % ^B,b^
Right lung	D_mean_ (Gy)	0.2 ± 0.1 ^A^	0.3 ± 0.2 ^A^	0.3 ± 0.1 ^A^	0.8 ± 0.4 ^B^
	V5	0.0 % ± 0.0 % ^A^	0.4 % ± 1.0 %	0.0 % ± 0.1 % ^a^	0.9 % ± 1.5 % ^B,b^
Right breast	D_mean_(Gy)	0.5 ± 0.3 ^A^	0.8 ± 0.4 ^B,a^	0.9 ± 0.3 ^B,a^	1.8 ± 0.8 ^B,b^
	V5	0.1 % ± 0.2 % ^A^	1.9 % ± 1.9 % ^B,a^	0.6 % ± 1.1 % ^B,b^	1.5 % ± 2.3 % ^B^
Esophagus	D_mean_(Gy)	0.3 ± 0.1 ^A^	0.4 ± 0.1 ^A^	0.4 ± 0.1 ^A^	0.7 ± 0.3 ^B^
Liver	D_mean_(Gy)	0.2 ± 0.1 ^A^	0.2 ± 0.1 ^A^	0.3 ± 0.2 ^A^	1.0 ± 0.7 ^B^
Spinal cord	D_mean_(Gy)	0.1 ± 0.0 ^A^	0.2 ± 0.0 ^A^	0.2 ± 0.0 ^A^	0.4 ± 0.2 ^B^

“A” is statistically significantly different from “B” (*p* < 0.05); “a” is statistically significantly different from “b” (*p* < 0.05). No other statistically significant difference was found between any two (*p* > 0.05)

The D_mean_ and V_Gy_ (the volume of the organ receiving at least a given dose (Gy)), for the left and right lungs, contralateral breast, esophagus, spinal cord and liver in the different treatment techniques are shown in Table 3. For left lung, the D_mean_ was similar for two IMRT and 2-arc VMAT, but worse for 1-arc VMAT. The 2-F IMRT plan reduced the volumes receiving low dose (V5), but increased the volumes receiving high doses (V40) compared with other plans. However, the 1-arc VMAT plan showed better results than other plans for left lung at V40 with statistical significance. The difference of V5-V40 between the 2-arc VMAT and the 4-F IMRT plan was not statistically significant.

Compared with the other plans for the other OARs, the 1-arc VMAT plan significantly increased the D_mean_ and V5 of the right lung and breast, and also the D_mean_ of esophagus, liver and spinal cord. Among the 2-F IMRT, 4-F IMRT and 2-arc VMAT plan, there was no statistically significant difference in these organs. Detailed results are given in Table 3.

### Dose analysis of heart and coronary arteries

Comparisons for the relevant dosimetric parameters for heart and coronary artery are given in Table 4. The mean dose to the entire heart was smaller for the IMRT plans compared with VMAT plans, but the mean dose to the coronary artery (CA) was similar in all plans. For the heart and CA, the 2-F IMRT plan reduced the V5 compared with other plans. The 1-arc VMAT plan was worse for V5, but showed good result for V20 and V40 compared with other plans. The 2-arc VMAT plan slightly increased the V20 and V40, but no difference was seen compared with the IMRT plans. For dose comparison of the region of interest: entire heart minus coronary artery, the V20 in all the plans was less than 1 % and not significantly different between any two.

**Table 4:** Dose comparison of the heart, coronary artery and heart minus coronary artery in the four plans

Structure	Dose parameter	2FIMRT	4FIMRT	2ArcVMAT	1ArcVMAT
Heart	D_mean_(Gy)	2.8 ± 1.0 ^A^	3.0 ± 1.4 ^a^	3.3 ± 1.3 ^B^	3.7 ± 1.4 ^B,b^
	D_max_(Gy)	50.2 ± 2.3 ^A^	48.2 ± 3.7 ^B,a^	44.1 ± 15.7	45.4 ± 5.2 ^B,b^
	V5	8.6 % ± 3.8 % ^A^	11.0 % ± 6.9 % ^a^	13.7 % ± 7.0 % ^B^	16.6 % ± 9.2 % ^B,b^
	V20	3.4 % ± 1.7 % ^A^	3.3 % ± 2.3 % ^A^	3.7 % ± 2.4 % ^B^	3.4 % ± 2.6 % ^A^
	V40	0.9 % ± 0.5 % ^A^	0.7 % ± 0.5 % ^B^	0.9 % ± 0.6 % ^a^	0.4 % ± 0.5 % ^B,b^
CA	D_mean_(Gy)	13.2 ± 3.9	13.3 ± 4.0	12.7 ± 5.8	12.7 ± 4.3
	D_max_(Gy)	50.2 ± 1.7 ^A^	48.7 ± 3.7 ^A^	48.9 ± 5.3 ^A^	45.6 ± 4.9 ^B^
	V5	56.4 % ± 15.4 % ^A^	60.8 % ± 16.6 %	62.1 % ± 18.6 % ^B^	62.7 % ± 15.2 % ^B^
	V20	26.1 % ± 10.5 %	25.7 % ± 10.9 %	27.2 % ± 12.3 % ^A^	24.8 % ± 13.3 % ^B^
	V40	7.3 % ± 3.8 % ^A^	5.9 % ± 3.5 % ^A^	6.8 % ± 4.0 % ^A^	3.3 % ± 3.5 % ^B^
Heart-CA	D_mean_(Gy)	1.4 ± 0.5 ^A^	1.8 ± 0.9 ^B,a^	2.0 ± 0.9 ^B,a^	2.5 ± 1.0 ^B,b^
	V5	2.4 % ± 2.0 % ^A^	5.6 % ± 5.3 % ^B,a^	7.3 % ± 6.1 % ^B^	10.5 % ± 8.5 % ^B,b^
	V20	0.3 % ± 0.6 %	0.6 % ± 1.2 %	0.6 % ± 1.2 %	0.6 % ± 1.1 %
	V40	0.1 % ± 0.2 %	0.1 % ± 0.2 %	0.1 % ± 0.2 %	0.0 % ± 0.1 %

“A” is statistically significantly different from “B” (*p* < 0.05); “a” is statistically significantly different from “b” (*p* < 0.05). No other statistically significant difference was found between any two (*p* > 0.05)

### Plans of quality score

From the summary of scoring (Table 5), we found that 2-F IMRT had the highest scrores of 14 with 4 points from heart and CA, followed by the 2-arc VMAT plan (10 points) with none from heart and CA, and the 1-arc VMAT plan (8 points) with the 4 points from heart and CA, finally the 4-F IMRT plan (6 points) with 2 points from heart and CA. From the individual plan comparison (Table 6), we found that 2-F IMRT plan showed advantage on multiple indices for almost patients. And for heart and CA, the 2-F IMRT and 1-arc VMAT also showed equally “most appearance score” on indices (both 3 point) (Table 6). These results suggested that there might be a clinical advantage for using 2-F IMRT over other plans in overall consideration.

**Table 5:** Plan score table of the four treatment techniques

Structure	Dose Parameter	2 F-IMRT	4 F-IMRT	2Arc-VMAT	1Arc-VMAT
PTV	HI	0	0	1	1
	CI	0	0	1	1
	CN	0	0	1	1
	MU	1	0	0	0
	Time	1	0	0	0
Lung.L	D_mean_	1	0	1	0
	V5	1	0	1	0
	V20	0	0	0	0
	V40	0	0	0	1
Heart	D_mean_	1	1	0	0
	V5	1	0	0	0
	V20	1	1	0	1
	V40	0	0	0	1
Coronary artery	D_mean_	0	0	0	0
	V5	1	0	0	0
	V20	0	0	0	1
	V40	0	0	0	1
Healthy tissue	D_mean_	1	1	1	0
Breast.R	D_mean_	1	0	0	0
Liver	D_mean_	1	1	1	0
P-cord	D_mean_	1	1	1	0
Eso	D_mean_	1	1	1	0
Lung.R	D_mean_	1	0	1	0
Total Score		14	6	10	8
Scores from Heart and CA		4	2	0	4

**Table 6:** Individual plan comparison table

Structure	Parameter	1	2	3	4	5	6	7	8	9	10	11	Most Appearance
PTV	HI	2A	2A	4 F	2A	2A	1A	1A	1A	1A	2A	2A	2A
	CI	1A	1A	1A	1A	1A	1A	1A	1A	1A	1A	1A	1A
	CN	1A	1A	1A	1A	1A	1A	1A	1A	1A	1A	1A	1A
	MU	2 F	2 F	2 F	2 F	2 F	2 F	2 F	2 F	2 F	2 F	2 F	2 F
	Time	2 F	2 F	2 F	2 F	2 F	2 F	2 F	2 F	2 F	2 F	2 F	2 F
Lung.L	D_mean_	2 F	2 F	2 F	2 F	2 F	2 F	2A	2 F	2 F	1A	4 F	2 F
	V5	2 F	2 F	2 F	2 F	2 F	2 F	2A	2A	2 F	1A	4 F	2 F
	V20	2 F	2 F	2A	2 F	2 F	1A	1A	2A	1A	1A	4 F	2 F /1A
	V40	4 F	1A	1A	4 F	1A	1A	1A	2A	4 F	1A	1A	1A
Heart	D_mean_	4 F	2 F	2 F	2 F	2 F	2 F	4 F	2A	4 F	1A	2 F	2 F
	V5	2 F	2 F	2 F	2 F	2 F	2 F	4 F	2A	2 F	2 F	2 F	2 F
	V20	4 F	4 F	4 F	2 F	4 F	4 F	4 F	2A	4 F	1A	1A	4 F
	V40	1A	1A	1A	1A	4 F	1A	1A	1A	4 F	1A	1A	1A
CA	D_mean_	4 F	4 F	1A	2 F	4 F	1A	1A	2A	4 F	1A	1A	1A
	V5	2 F	2 F	4 F	2 F	2 F	4 F	2 F	2A	4 F	1A	4 F	2 F
	V20	4 F	4 F	4 F	2 F	4 F	4 F	1A	2A	4 F	1A	1A	4 F
	V40	4 F	1A	1A	4 F	1A	1A	1A	1A	4 F	1A	1A	1A
Healthy tissue	D_mean_	2 F	2 F	2 F	2 F	2 F	2 F	2 F	2A	2 F	2A	2 F	2 F
Breast.R	D_mean_	2 F	2 F	2 F	2 F	2 F	4 F	2 F	2 F	2 F	2 F	2 F	2 F
Liver	D_mean_	2A	2A	2 F	2 F	2 F	4 F	2 F	2A	2 F	4 F	2 F	2 F
P-cord	D_mean_	2 F	2 F	2 F	2 F	2 F	2 F	2 F	2 F	2 F	4 F	2 F	2 F
Eso	D_mean_	2 F	2 F	2 F	2A	2 F	2 F	2 F	2A	2 F	2 F	2 F	2 F
Lung.R	D_mean_	2 F	2 F	2 F	2 F	2 F	2 F	2 F	2 F	2 F	2 F	2 F	2 F
Most Appearance		2 F	2 F	2 F	2 F	2 F	2 F	2 F/1A	2A	2 F	1A	2 F	

*Abbreviations*: *1A* 1-arc VMAT, *2A* 2-arc VMAT, *2 F* 2-F IMRT, *4 F* 4-F IMRT

## Discussion

Planning comparisons and dosimetric studies of different field IMRT or VMAT in breast cancer have been evaluated in a large number of studies and there’s always been a debate on employing which technique in the radiation practice. This study compares different arcs of VMAT and fields of IMRT in radiotherapy planning, and evaluates the plans with the quality score table which focused on heart dose and coronary area in left sided breast cancer radiotherapy. However, the advantage of suitable radiotherapy plan for the patients with relative smaller breast has not been fully clarified.

Patients with early stage left breast cancer could survive for a long time and adapt to receive techniques that may reduce the incidence of acute and late toxicity induced by radiotherapy. The cardiovascular complications induced by radiation- as a main radiotherapy-related late toxicity event progresses over time, and may manifest decades after the initial exposure [19]. The coronary artery injury was considered to be the most serious radiation-related complication in the heart. Darby SC et al*.* reported that exposure of the heart to ionizing radiation during radiotherapy for breast cancer increases the subsequent rate of ischemic heart disease linearly with the mean dose to the heart by 7.4 % per gray, with no apparent threshold [20]. Other studies further suggested that 1 Gy irradiation added to the mean heart dose could increase the cardiotoxic risk by 4 % [21]. Several studies had observed substantial radiation-induced heart disease when the heart receives more than 40 Gy and that the reduction of the V40 was pertinent in reducing heart toxicities [22, 23]. In the present study, when calculated the scores from the sections of heart and coronary arteries, we found that both the 1-arc VMAT and 2-F IMRT have the highest scores of 4 points. The former showed advantage on V20 and V40 for heart and coronary arteries, and the latter showed favorable results on D_mean_, V5 and V20 for heart and V5 for coronary arteries. This meant the 2-F IMRT and 1-arc VMAT plan showed a statistically significant improvement for heart dose for left-sided breast irradiation.

Nowadays, the developed radiation techniques could also be used to spare the cardiac area sparing in breast radiation practice. It was reported that radiation delivering in Deep Inspiration Breath Hold (DIBH) conditions could reduce the dose to heart for left-sided breast cancer patients [24]. Some other studies demonstrated that whole breast irradiation with prone position seems to be beneficial for 85 % of the patients regarding heart irradiation [25]. Further studies found that IMRT with prone position is superior to supine treatment for right-sided breast cancer patients and left-sided breast cancer patients with larger breasts [26] and benefited most from prone position with DIBH for heart sparing by radiation dose [27]. But for patients of smaller breast volume in left side, some studies argued that the prone position might result in worse cardiac dosimetry than supine position [28, 29].

Moreover, when a dose comparison of heart minus coronary artery calculated, the observed V20 and V40 for the rest of heart, in all plans were very small and closed, suggesting that the dose volume for the coronary arteries can be used to predict the dose volume of the high dose for the entire heart.

For other OARs, it has been reported that the doses to the ipsilateral lung have been shown to be responsible for radiation pneumonia in breast cancer radiotherapy [30]. Dosimetric parameters of mean lung dose and V20 showed a significant correlation with the development of radiation-induced pneumonitis in radiotherapy for breast cancer [31, 32]. In our study, we found that for D_mean_ and V5 to ipsilateral lung, the best results were from 2-F IMRT and 2-arc VMAT plans, and the V20 to the ipsilateral lung was uneventful in all plans. In addition, the 2-F IMRT, 4-F IMRT and 2-arc VMAT plans were associated with the most favorable dose deposition in the liver, esophagus, spinal cord, contralateral lung compared with 1-arc VMAT.

Except for acute and late radiation damage induced by high dose radiation, the low dose irradiation raises the concern of radiation-induced secondary malignancy [33]. The delivery of low-dose irradiation to healthy tissue, especially to the contralateral breast, has been estimated to double the risk of subsequent malignancy [34], and this risk increases with increasing dose [15]. Based on our study, it was demonstrated that 2-F IMRT and 2-arc VMAT resulted in a reduction of the mean dose to healthy tissue and ID as compared with that in other plans. And the 2-F IMRT plan also showed advantage on D_mean_ in contralateral breast.

So from the overall consideration we suggest to choose 2-F IMRT with the highest scores which was suitable for the protection of heart and coronary artery in left-sided breast cancer radiotherapy. We also found that 2-arc VMAT technique with the second highest scores could improve the homogeneity and conformity in PTV and sparing of some OARs in some dosimetric indications. So if the doctor has not concerns on the heart and coronary artery, the 2-arc VMAT technique may also be a good choice. The 1-arc VMAT plan with fewer composite scores has apparent advantages on D_max_ and high doses regions to heart and coronary artery, which might also be a selective plan for sparing heart dose in practice. The 4-F IMRT plans did not show special advantages when compared with other plans in our research.

## Conclusion

In conclusion, compared with other plans, the 2-F IMRT plan has demonstrated the combined advantages in PTV dose coverage and dose drop to most normal tissue involved in our research, besides for the heart and coronary artery. So we suggest employing 2 F-IMRT plan for left breast cancer radiotherapy after breast-conserving surgery.

### Consent

Written information consent was obtained from the patient for publication of this report and any accompanying images.

## Abbreviations

CA: coronary artery
CTV: clinical target volume
IMRT: intensity-modulated radiation therapy
OARs: organs at risk
PTV: planning target volume
VMAT: volumetric-modulated arc therapy
